# Description of *Culicoides* (*Culicoides*) *bysta* n. sp., a new member of the Pulicaris group (Diptera: Ceratopogonidae) from Slovakia

**DOI:** 10.1186/s13071-017-2195-4

**Published:** 2017-06-02

**Authors:** Adela Sarvašová, Alica Kočišová, Ermanno Candolfi, Bruno Mathieu

**Affiliations:** 1 Department of Parasitology, University of Veterinary Medicine and Pharmacy in Košice, Komenského 73, SK-04181 Košice, Slovak Republic; 2 Medicine Faculty, Institute of Parasitology and Tropical Pathology (IPPTS), EA7292, 3 rue Koeberlé, F-67000 Strasbourg, France; 3 Laboratoire de Parasitologie et de Mycologie Médicale, Hôpitaux Universitaires de Strasbourg, F-67000 Strasbourg, France

**Keywords:** *Culicoides*, Pulicaris group, DNA barcodes, New species description, *Culicoides bysta* n. sp.

## Abstract

**Background:**

Species of the genus *Culicoides* Latreille, 1809 (Diptera: Ceratopogonidae) are mainly known as vectors of arboviruses such as bluetongue (BTV) and Schmallenberg (SBV). Among the known vectors, few species within the subgenus *Culicoides* Latreille, 1809 have been implicated in the transmission of BTV and SBV. Nevertheless, phylogenetic studies had revealed the presence of cryptic and undescribed species in Europe, raising questions about their vectorial role. A previous integrative study, associating morphology and barcode data, raised the hypothesis of the presence of undescribed species in Slovakia. The present study, combining morphological and molecular approaches, is aimed to support the hypothesis and a description of *Culicoides bysta* n. sp. is provided.

**Methods:**

Series of male and female specimens were dissected and several of them were sequenced for the barcode region of the mitochondrial cytochrome *c* oxidase subunit 1 gene (*cox*1). Bayesian inference phylogenetic analyses based on 72 *cox*1 sequences of the species belonging to the Pulicaris group of the subgenus *Culicoides*, were carried out and the frequencies of intra/interspecific variations were analyzed. The morphology of abundant material of the new species (31 females and 12 males) was examined and compared with the paratypes of *Culicoides boyi* Nielsen, Kristensen & Pape, 2015 and with specimens of *Culicoides pulicaris* Linnaeus, 1758. For females, suture distances on the eyes were newly evaluated as a diagnostic character and for males we assessed a new measurement on the ninth tergite and on the apicolateral processes.

**Results:**

Both phylogenetic analysis and barcode distances supported the distinct status of the new species, *Culicoides bysta* n. sp. described as a member of the Pulicaris group based on the morphology of males and females. The new species is closely related to *C. boyi* and *C. pulicaris* but can be distinguished on the basis of the wing pattern and the ratio between the two eye sutures. Both newly evaluated characters, i.e. eyes in females and male genitalia appeared to be diagnostic for distinguishing the new species described herein.

**Conclusions:**

The vector potential of the recently described species *C. boyi* and *C. bysta* n. sp. to transmit arboviruses, such as BTV and SBV, is unknown. When considering these two species as being close to *C. pulicaris*, the previous data, such as the vector implication for *C. pulicaris* in BTV transmission, should be revaluated in future.

## Background

The biting midges of the genus *Culicoides* Latreille, 1809 (Diptera: Ceratopognidae) are small hematophagous insects. The biodiversity of this genus represented more than 1300 species worldwide [1] including some species implicated as vectors of arboviruses. In Europe, the species belonging to the subgenera *Avaritia* Fox, 1955 and *Culicoides* have been pointed out as potential vectors, at various levels, of the Bluetongue virus (BTV): *C.* (*Avaritia*) *obsoletus* (Meigen, 1818)*/ C.* (*A.*) *scoticus* Downes & Kettle, 1952; *C.* (*A.*) *dewulfi* Goetghebuer, 1936; *C.* (*A.*) *chiopterus* (Meigen, 1830); *C.* (*A.*) *imicola* Kieffer, 1913; *C.* (*Culicoides*) *pulicaris* (L.); and *C.* (*C.*) *lupicaris* Downes & Kettle, 1952 [2–10]. Within the subgenus *Culicoides*, *C.* (*C*.) *punctatus* (Meigen, 1804), a species close to *C. pulicaris*, has recently been mentioned as participating in the transmission of the Schmallenberg virus [11].

The exact number of species belonging to the subgenus *Culicoides* in the Palaearctic region is unknown, as different authors include different species in the subgenus [12]. Other authors presented their disagreement regarding the subgeneric nomenclature and thus classified the species related to *C. pulicaris* as the species of the Pulicaris group [13] and eventually subgroups [14]. Regardless of the chosen classification, the species related to and “grouped” with *C. pulicaris* vary among the authors. In an attempt to clarify the classification and the identification of the species within the Pulicaris group, phylogenetic studies based on *cox*1 mitochondrial gene sequences [15–19], as well as on ITS2 rDNA region [12, 20], revealed the presence of cryptic species. Following the discovery of this genetic diversity, in 2013, a new species, *C. paradoxalis* Ramilo & Delécolle, 2013, close to *C. lupicaris* was described from France and Portugal [21]. In Denmark, three species molecularly characterised in 2011 [18], have been recently described as new species, i.e. *C. boyi*, *C. selandicus* Nielsen, Kristensen & Pape, 2015 and *C. kalix* Nielsen, Kristensen & Pape, 2015 [18, 22]. It is worth to mention that for the four above mentioned recently described species, i.e. the one from Portugal and the three from Denmark, morphological studies have highlighted the diagnostic characters for accurate identification [21–23]. In the light of the cryptic diversity detected in this group, further undescribed species may be expected [15, 17, 19]. Further studies, including morphological exploration, are still required to eventually lead to the formal description of new species. Currently, new species are frequently discovered by both genetic discrepancies detected by barcode analyses (*cox*1 region) and the presence of highly supported clade from phylogenetic studies [21, 22]. Besides, morphological studies are highly recommended to be linked to these genetic studies, for a better understanding of the composition of subgenus *Culicoides* [19].

In the present study, we describe a member of the Pulicaris group, *C.* (*Culicoides*) *bysta* n. sp. The sample consisting of a unique female specimen found in our previous study [19] is now completed with a larger series of males and females. To support the distinct species status of the new species, we implemented a combination of morphological and phylogenetic (based on *cox*1 gene) approaches.

## Methods

### Sampling and identification of *Culicoides*

Specimens used in this study were collected at 3 permanent trapping sites in eastern Slovakia (game park in Rozhanovce and cattle farms in Michalany and Tulcik), where the CDC miniature light trap model 1212 (John Hock Company, USA) was operated weekly, from April to November 2011–2015. In addition, occasional collections were done across Slovakia: Bysta (game park), Pcoline (cattle farm), Velaty (farm with different animals), Antalka (family house with animals), Ziar (family weekend house with sheep). The collection and identification procedures were described in the previous paper [19]. Specimens from Bulgaria, Kosovo, and Denmark were collected and identified within the VectorNet project, whereas the ones from France were from the French surveillance network funded by the French Ministry.

**Fig. 1:**
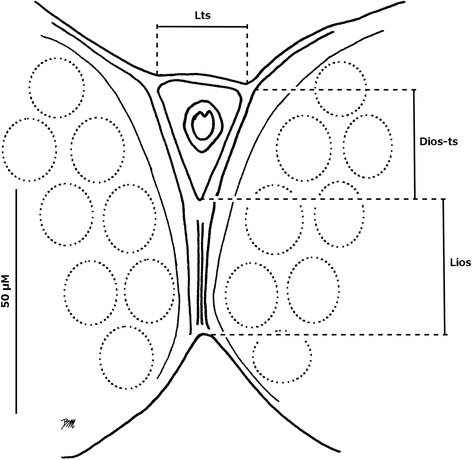
Schematic illustration of joined eyes of a *Culicoides* spp. female, showing the measurements. *Abbreviations*: Lios, length of the inter-ocular suture; Lts, length of the transverse suture; Dios-ts, distance between the inter-ocular suture and the transverse suture

**Fig. 2:**
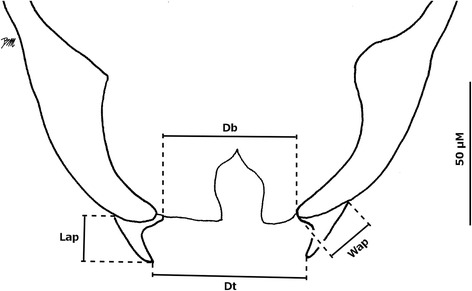
Ninth tergite of male *Culicoides* spp*.* The measurements of the length (Lap) and the width (Wap) of the apical processes, the distance separating the two tips of the apical processes (Dt) and the distance between the bases of the two apical processes (Db) are shown

Morphological terminology follows Mathieu et al. [24] and Sarvašová et al. [19]. The antennal trichodea ratio (AtR), described by Meiswinkel [25], was measured for females to evaluate its diagnostic potential within the Pulicaris group. The ratio of the third segment, calculated by dividing the length of the first flagellomere by its width, previously evaluated by Nielsen et al. [22], was measured for females. On the eyes we investigated the usefulness of the new characters illustrated in Fig. 1. The length of the inter-ocular suture of the joined eyes (Lios) and the distance between the latter and the transverse suture above the first inter-ocular seta (Dios-ts) were measured. Subsequently, we computed the ratio consisting in Lios divided by Dios-ts; the length of the transverse suture was also reported (Lts). In order to discriminate males from the Pulicaris group, new morphological characters of aedeagus were measured and analyzed (Fig. 2): the length (Lap) and width (Wap) of the apical processes of the ninth tergite, the distance separating the two tips of the apical processes (Dt), and the distance separating the base of the two apical processes (Db). All measurements are in micrometres and are provided as the mean followed by the range (minimum-maximum) and the number of measurements in parentheses. The difference between measurements was determined by Mann-Whitney test (*P* < 0.05) using R software [26]. Characters measured for more than two groups were tested by Kruskal-Wallis rank sum test prior to Mann-Whitney.

The holotype and 10 paratypes are deposited in the collection of the Institut de Parasitologie et de Pathologie Tropicale de Strasbourg (IPPTS), 10 paratypes in the Slovak National Museum and 11 paratypes are deposited in the collection of the University of Veterinary Medicine and Pharmacy in Košice.

### DNA barcode and phylogenetics

The following 53 *cox*1 (mtDNA) sequences have been gathered from the Genbank: *C. boyi* (JF766293-96), *C. bysta* n. sp. (KJ624118) referred as *speA* in a previous study [19], *C. kalix* (JF766328-31), *C. lupicaris* (HQ824431-33, KJ624097), *C. newsteadi* Austen GB (AM236742-46), *C. newsteadi* N1 (GQ338915, KJ624101), *C. newsteadi* N2 (GQ338916-20), *C. newsteadi* N3 (GQ338921-22, JF766322-23, JF766327), *C. pulicaris* P1 (AM236714-15, GQ338912-14, HQ824422-23, JF766363, JF766365, KJ624116-17), *C. pulicaris* P3 (GQ338910-11, KF591611), *C. punctatus* (JF766325-26, JF766356, JQ620186-88, KJ624119), and *C. selandicus* (JF766320, JF766324).

In addition, we extracted the DNA from thorax and first abdominal segments of 17 specimens of *C. bysta* n. sp. and two *C. boyi,* using the DNeasy blood and tissue kit (Qiagen, USA)*.* At the start of this study, barcoding fragments of the first 11 specimens have been amplified following the protocol of Pagès et al. [15] using the primers C1J1718/C1N2191 [27]. The resulting *cox*1 sequence size of 472 bp is similar to the most *cox*1 sequences available for *Culicoides* species. To produce longer sized amplicons allowing more complete genetic studies, the *cox*1 of the last 8 specimens have been amplified using the primer pairs LCO1490/HCO2198 [28] allowing a sequence size of up 687 bp. PCR products have been purified and sequenced by the Eurofins MWG Operon (Ebersberg, Germany). Information associating the origin and the accession number of the 19 newly sequenced specimens are presented in Table 1.

**Table 1:** Collection data for specimens of *Culicoides bysta* n. sp. and *C. boyi* used for genetic analyses

GenBank ID^a^	Species	Sex	Collection date	Country	Locality	Coordinates
KY436038	*C. bysta* n. sp.	F	14 July 012	Slovakia	Bysta	48°31'N, 21°33'E
KY436039	*C. bysta* n. sp.	F	11 May 2013	Slovakia	Michalany	48°30'N, 21°37'E
KY436040	*C. bysta* n. sp.	M	8 August 2013	Slovakia	Rozhanovce	48°45'N, 21°21'E
KY436041	*C. bysta* n. sp.	M	31 July 2013	Slovakia	Rozhanovce	48°45'N, 21°21'E
KY436042	*C. bysta* n. sp.	F	26 June 2014	Slovakia	Pčoline	49°03'N, 22°10'E
KY436043	*C. bysta* n. sp.	F	16 May 2013	Slovakia	Tulcik	49°5'N, 21°18'E
KY436044	*C. bysta* n. sp.	F	19 June 2012	France	Longeville-en-Barois	48°44'N, 5°13'E
KY436045	*C. bysta* n. sp.	F	19 June 2012	France	Longeville-en-Barois	48°44'N, 5°13'E
KY436046	*C. bysta* n. sp.	F	19 June 2012	France	Longeville-en-Barois	48°44'N, 5°13'E
KY436047	*C. bysta* n. sp.	F	19 June 2012	France	Longeville-en-Barois	48°44'N, 5°13'E
KY436048	*C. bysta* n. sp.	F	19 June 2012	France	Longeville-en-Barois	48°44'N, 5°13'E
KY436049	*C. bysta* n. sp.	F	26 June 2015	France	Crastatt	48°39'N, 7°25'E
KY436050	*C. bysta* n. sp.	F	17 July 2015	France	Jetterswiller	48°40'N, 7°25'E
KY436051	*C. bysta* n. sp.	F	25 June 2015	France	Rangen	48°40'N, 7°28'E
KY436052	*C. bysta* n. sp.	F	21 August 2015	Bulgaria	Muldava	41°59'N, 24°56'E
KY436053	*C. bysta* n. sp.	F	21 August 2015	Bulgaria	Topolovo	41°54'N, 25°01'E
KY436054	*C. bysta* n. sp.	F	1 September 2015	Kosovo	Stubel	42°20'N, 21°27'E
KY436055	*C. boyi*	F	26 June 2015	Denmark	Zealand	55°15'N, 12°01'E
KY436056	*C. boyi*	F	26 June 2015	Denmark	Zealand	55°15'N, 12°01'E

^a^GenBank accession numbers refer to cytochrome *c* oxydase 1 (*cox*1) sequences

The 72 above mentioned *cox*1 sequences were aligned using the ClustalW [29] and genetic distances were computed using the Jukes-Cantor model of MEGA version 6 [30]. The best-fit model of nucleotide substitutions was calculated as HKY + I+ Γ by the JModelTest v.2.1.4 [31]. The latter model was used to parameterise a phylogenetic analysis carried out under the Bayesian Inference (1,000,000 generations), using the MrBayes v3.1.2 [32]. Two-thousand and five-hundreds of the saved trees were discarded and the remaining 7500 trees were used to construct the phylogenetic tree. Clade posterior probabilities (CPP) estimates were used to assess the robustness of tree nodes.

## Results

### DNA barcode and phylogenetics

**Fig. 3:**
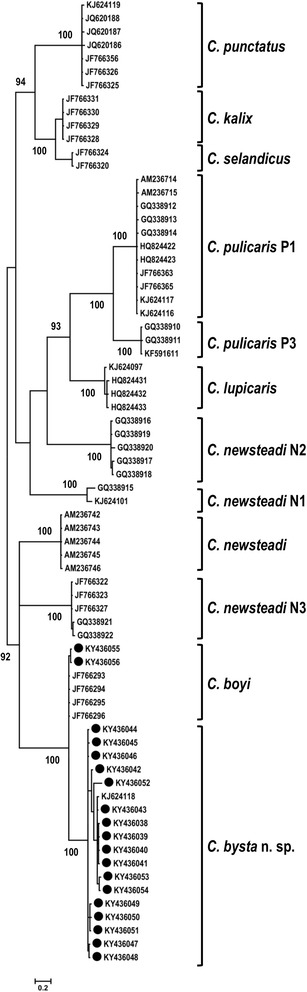
Unrooted Bayesian tree resulting from the phylogenetic analysis of 72 *cox*1 sequences. CPP values > 90% are included to indicate the branch support and *black circles* refer to sequences generated by this study

**Fig. 4:**
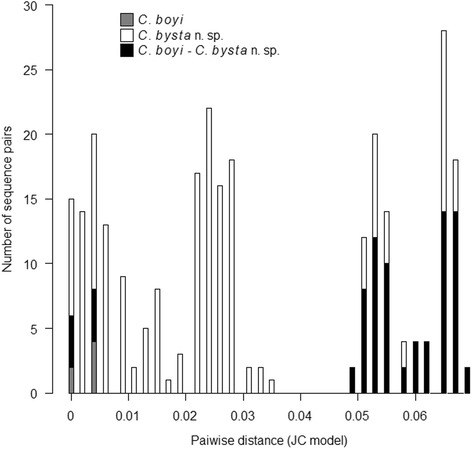
Frequences of the pairwise distances showing intra- (*grey and white*) and interspecific (*black*) variations

The phylogenetic tree obtained by the Bayesian Inference is shown in Fig. 3. All specimens within the species and the cryptic species pointed out by previous authors [15, 22] are gathered into highly supported clades (CPP = 100%). Three other nodes are quite well supported: (i) The node separating the three morphologically close species *C. punctatus*, *C. kalix,* and *C. selandicus* was well supported (CPP = 94%); (ii) the two cryptic species *C. pulicaris* P1-P3 and *C. lupicaris* clustered into a clade supported by the 93% CPP; and (iii) a clade supported by the 92% CPP included *C. newsteadi*, *C. newsteadi* N3, *C. bysta* n. sp. and *C. boyi*. For each species, intra- and interspecific genetic distances are shown in Table 2. *Culicoides newsteadi* N1 and *C. bysta* n. sp. showed a mean of intraspecific distances of 3.4 and 1.6%, respectively. With the exception of the two latter species, all species have the mean of intraspecific distances lower than 1%. The minimum interspecific distances are the lowest between *C. kalix/C. selandicus* and *C. bysta* n. sp./*C. boyi* with 7.1 and 5.6%, respectively. For all the other species, the minimum interspecific distances are higher than 11%. Focusing on *C. bysta* n. sp. and *C. boyi*, pairwise distances are computed and frequencies are plotted by intra and interspecific variations (Fig. 4). No overlaps between the intraspecific distances (intra-*C. boyi* and intra-*C. bysta* n. sp.) and the interspecific distances were shown.

**Table 2:** Intra- and interspecific genetic distances computed using the Juke and Cantor model in MEGA. Minimum (Min) and Maximum (Max) distance values are presented as percentages

Species	Intraspecific			Interspecific	
	Mean	Min	Max	Min	Max
*C. boyi*	0	0	0	5.6	23.9^a^
*C. bysta* n. sp.	1.6	0	3.4	5.6	24.6^a^
*C. kalix*	0	0	0	7.1	20.1
*C. lupicaris*	0.5	0.5	0.5	15.2	22.1
*C. newsteadi*	0.1	0	0.3	14.8	24.6
*C. newsteadi* N1	3.4	3.4	3.4	17.1	22.1
*C. newsteadi* N2	0.8	0.3	1.6	17.7	22.5
*C. newsteadi* N3	0.3	0	0.5	16.1	22.8
*C. pulicaris* P1	0.3	0	0.8	11.2	22.8
*C. pulicaris* P3	0.2	0	0.3	11.2	24.6
*C. punctatus*	0.4	0	1	13.3	22.8
*C. selandicus*	0.3	0.3	0.3	7.1	21.8

^a^Maximum interspecific distance between *C. bysta* n. sp. and *C. boyi* is 7.6%

### Taxonomy

**Family Ceratopogonidae, Newman, 1834**

**Genus** ***Culicoides*** **Latreille, 1809**

**Subgenus** ***Culicoides*** **Latreille, 1809**

***Culicoides*** **(** ***Culicoides*** **)** ***bysta*** **Sarvašová & Mathieu n. sp.**

Syn. *Culicoides* sp. of Sarvašová et al. [19] (referred to as *Culicoides* speA)

***Type-locality*** **:** Rozhanovce (48°45'N, 21°21'E), Slovakia.

***Other localities*** **:** Slovakia - Antalka, 48°21'N, 19°56'E, Bysta, 48°31'N, 21°33'E, Michalany, 48°30'N, 21°37'E, Pcoline, 49°03'N 22°10'E, Tulcik, 49°5'N, 21°18'E, France - Crastatt, 48°39'N, 7°25'E, Jetterswiller, 48°40'N, 7°25'E, Longeville-en-Barois, 48°44'N, 5°13'E, Rangen, 48°40'N, 7°28'E, Bulgaria - Muldava, 41°59'N, 24°56'E, Topolovo, 41°54'N, 25°01'E, and Kosovo - Stubel, 42°20'N, 21°27'E.

***Type-material*** **:** The holotype and 10 paratypes (acc. no. IPPT-CL-2017-01 to IPPT-CL-2017-11) are deposited in the collection of the Institut de Parasitologie et de Pathologie Tropicale de Strasbourg (IPPTS), 10 paratypes (acc. no. SZ10896 to SZ10905) in the Slovak National Museum, and 11 paratypes (acc. no. UVLF-CL-2017-01 to UVLF-CL-2017-11) are deposited in the collection of the University of Veterinary Medicine and Pharmacy in Košice as detailed below. Holotype male (IPPT-CL-2017-01): Slovakia: Rozhanovce, 48°45'N, 21°21'E, 11.vii.2013. Paratypes: Slovakia: Antalka, 48°21'N, 19°56'E, 7.vi.2014 (1 female, SZ10900); Bysta, 48°31'N, 21°33'E, 14.vii.2012 (1 female,UVLF-2017-01), 28.vii.2012 (1 female, SZ10898); Michalany, 48°30'N, 21°37'E, 25.v.2011 (1 female, SZ10897), 11.v.2013 (1 female;IPPT-CL-2017-03), 10.v.2014 (2 females, IPPT-CL-2017-04, IPPT-CL-08); Pcoline, 49°03'N, 22°10'E, 26.vi.2014 (1 female, IPPT-CL-2017-02); Rozhanovce, 48°45'N, 21°21'E, 8.v.2013 (4 females, SZ10899, UVLF-CL-2017-03, UVLF-CL-2017-05, UVLF-CL-2017-06), 11.vi.2013 (1 male, UVLF-CL-2017-10), 31.vii.2013 (1 female, UVLF-CL-2017-02), 8.viii.2013 (1 male, IPPT-CL-2017-09), 31.vii.2013 (1 male, UVLF-CL-2017-08), 13.viii.2013 (2 females; IPPT-CL-2017-07, UVLF-CL-2017-07), 11.v.2014 (1 male, UVLF-CL-2017-11), 3.ix.2014 (1 female, IPPT-CL-2017-05, 6 males, SZ10902, SZ10903, SZ10904, SZ905, IPPT-CL-2017-10, IPPT-CL-2017-11, UVLF-CL-2017-09), 21.viii.2014 (2 female, SNMxx, IPPT-CL-2017-06;); Tulcik, 49°5'N, 21°18'E, 16.v.2013 (3 females; SZ10896, SZ10901, UVLF-CL-2017-04).

***Non-type material examined*** **:** France: Crastatt, 48°39'N, 7°25'E, 26.vi.2015 (1 female); Jetterswiller, 48°40'N, 7°25'E, 17.vii.2015 (1 female); Longeville-en-Barois, 48°44'N, 5°13'E, 19.vi.2012 (5 females); Rangen, 48°40'N, 7°28'E, 25.vi.2015 (1 female). Bulgaria: Muldava, 41°59'N, 24°56'E, 21.viii.2015 (1 female); Topolovo, 41°54'N, 25°01'E, 21.viii.2015 (1 female). Kosovo: Stubel, 42°20'N, 21°27'E, 1.ix.2015 (1 female). The non-type material is deposited in the collection of the IPPTS under the accession number IPPT-CL-2017-00.

***ZooBank registration*** **:** To comply with the regulations set out in article 8.5 of the amended 2012 version of the *International Code of Zoological Nomenclature* (ICZN) [33], details of the new species have been submitted to ZooBank. The Life Science Identifier (LSID) of the article is urn:lsid:zoobank.org:pub:0C1AA32E-9C07-402B-BB58-7B5BFF72EA19. The LSID for the new name *Culicoides bysta* is urn:lsid:zoobank.org:act:BFB32DDD-C44C-40D9-8427-EAF869F122C0.

***Etymology*** **:** Named after a small village in south-eastern Slovakia, where the species was detected for the first time.

### Description

**Fig. 5:**
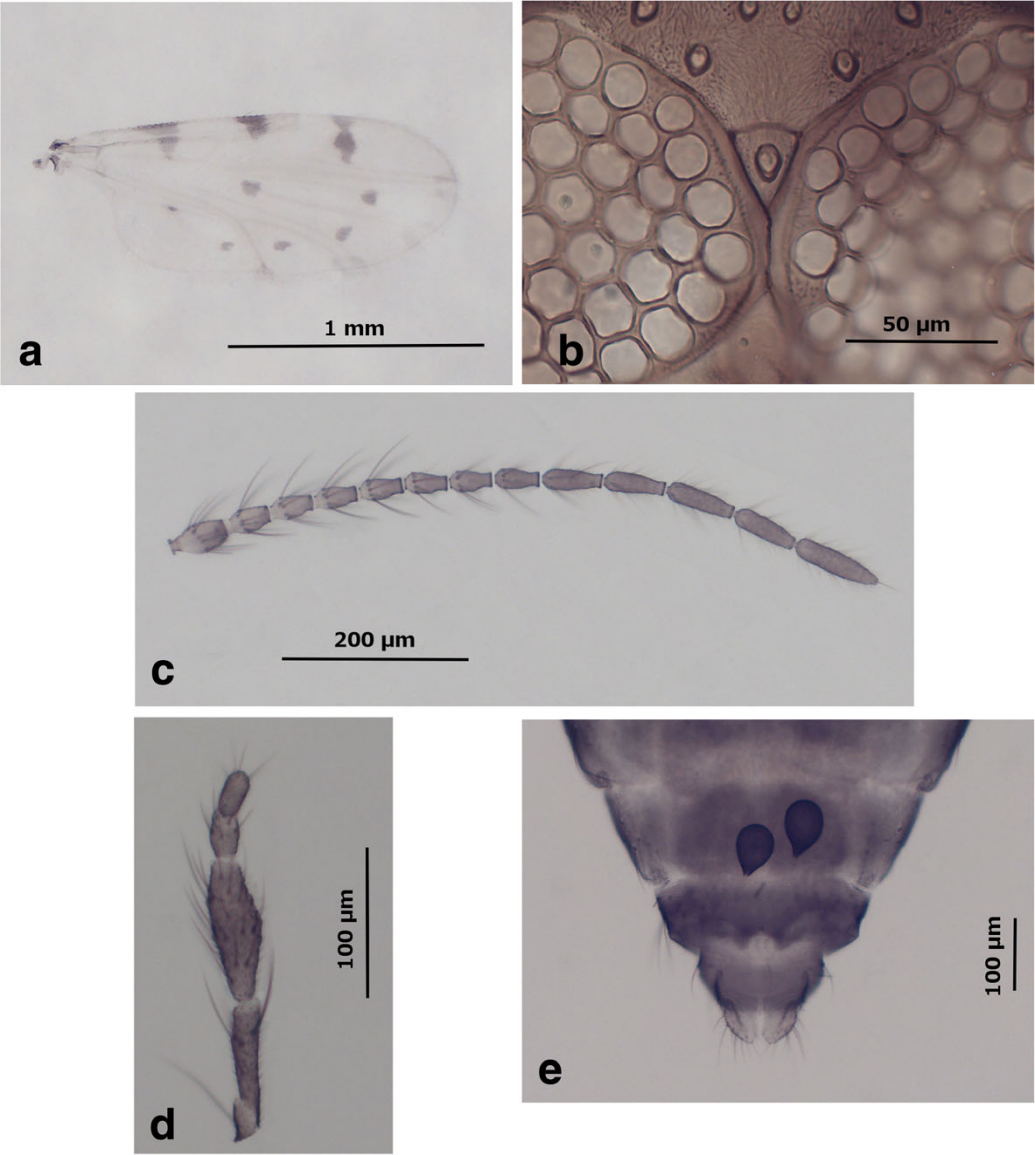
Images of *C. bysta* n. sp. female. **a** Wing. **b** Eyes. **c** Antenna. **d** Palpus. **e** Last abdominal segments with spermathecae. *Scale-bars*: **a**, 1 mm; **b**, 50 μm; **c**, 200 μm; **d**, **e**, 100 μm

**Fig. 6:**
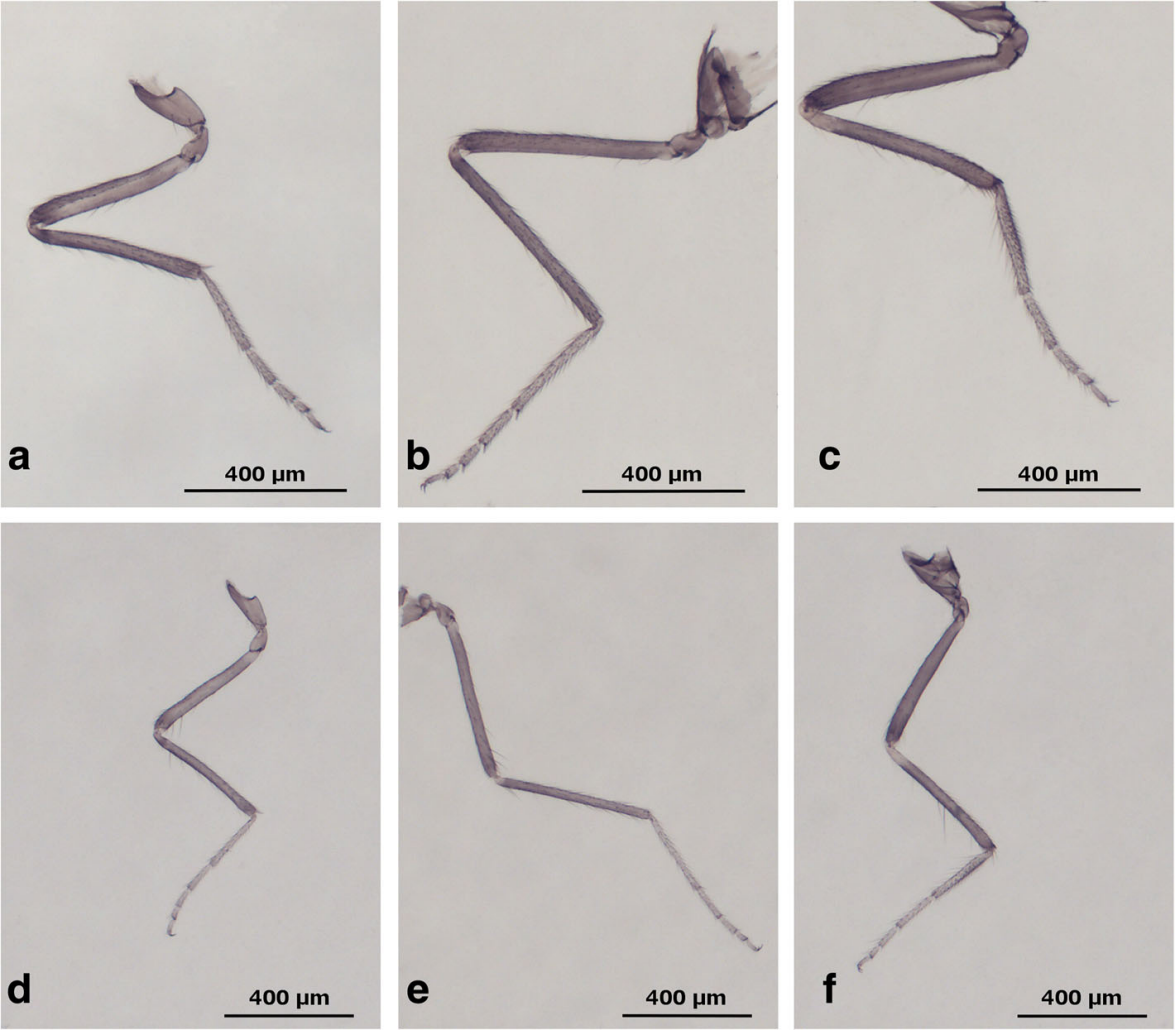
Images of legs of *C. bysta* n. sp. **a**-**c** Fore, middle and hindleg of a female, respectively. **d**-**f** Fore, middle and hindleg of a male, respectively. *Scale-bars*: 400 μm

**Fig. 7:**
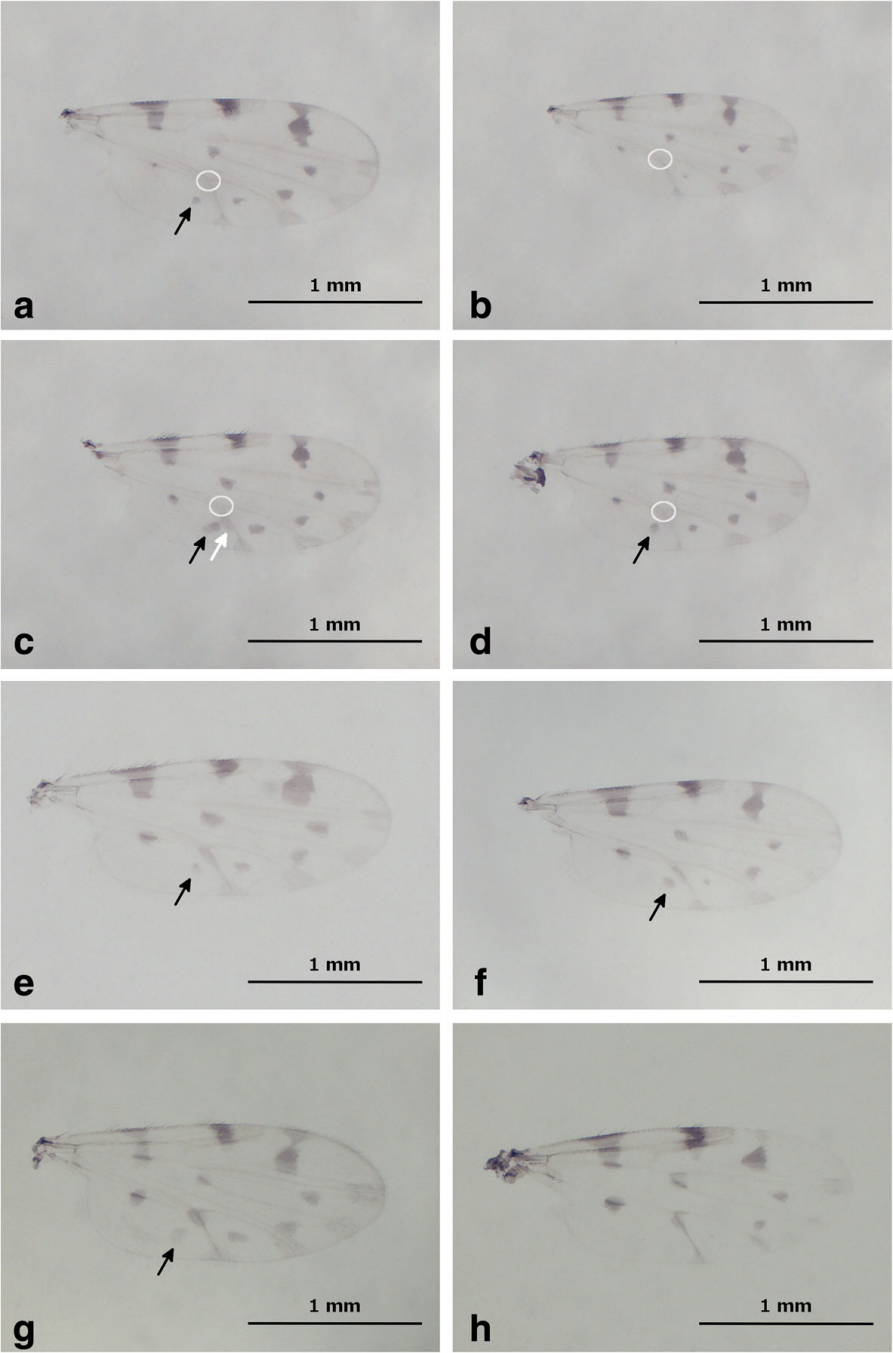
Images showing wing pattern variations within the *C. bysta* n. sp., *C. boyi* and *C. pulicaris.* **a**-**d** *C. bysta* n. sp. **e**-**f** *C. boyi*. **g**-**h** *C. pulicaris*. Light circles show the pale cubital-anal fork while the circle absence means a dark fork. *Black* and *white arrows* indicate the regular and the extra *dark spot* in the anal cell, respectively. *Scale-bars*: 1 mm

***Adult female*** *.* [Based on 20 paratypes; Figs. 5, 6a-c, and 7a-d.] *Head*: Eyes (Fig. 5b) bare, contiguous over a distance which can be estimated as approximately the diameter length between one and two adjacent facets, 34.8 (21.9–46.0, *n* = 20). Antenna (Fig. 5c): sensilla coeloconica present on flagellomeres 1, 9–13 (respective sensilla numbers are presented in Table 3); blunt-tipped sensilla trichodea distributed as 2 long on flagellomere 1, 2 long and 1 short on segments 2–8; AtR 1.50 (1.15–1.87, *n* = 40); length of flagellomeres 1–8 347 (297–400, *n* = 33); total length of flagellum 719 (647–792, *n* = 22); antennal ratio 1.03 (0.92–1.16, *n* = 22); R11/10 given in Table 4; ratio of first flagellomere 1.64 (1.3–1.8, *n* = 25). Palpus (Fig. 5d) slender, palpal segment I with 1 long chaetica; segment II with 3.9 chaeticae; segment III slightly swollen, carrying 13.1 chaeticae, with multiple irregular pits sparse on segment; segment IV with 7 short chaeticae; segment V without chaetica but with 5 apical bristles (*n* = 10); length of segments I–V: 87.2 (76–101) (I + II), 90.6 (78–106), 34.9 (25–44) and 36.4 (32–44); total length 250 (216–287, *n* = 25); PR 2.7 (2.4–3.1, *n* = 39) (proboscis length and P/H ratio are presented in Table 4). Maxilla with 18 (17–21, *n* = 10) teeth; mandible with 14.36 (12–16, *n* = 33) teeth. Cibarial and pharyngeal armature absent. *Thorax*: Legs (Fig. 6a–c) brownish, with usually pale bands described hereafter. Foreleg: proximal part of femur slightly pale, fore-tarsus pale, spines on all tarsal segments absent; lengths of femur, tibia and tarsal segments: 417, 432, 226, 106, 69, 39 and 44 (*n* = 5); foreleg TR 2.1 (*n* = 5). Middle leg: knee and mid-tarsus pale, 2 spines present distally on first tarsal segments, 2 on second, 2 on third, 1 on fourth; lengths of femur, tibia and tarsal segments: 543, 548, 288, 110, 69, 43 and 48 (*n* = 5); middle leg TR 2.6 (*n* = 5). Hind leg: pale ring on proximal part of hind-tibia, tibial comb with 5.9 (5–7, *n* = 36) spines, spines on all tarsal segments absent; lengths of femur, tibia and tarsal segments: 515, 524, 273, 146, 81, 47 and 58 (*n* = 5); hind leg TR 1.9 (*n* = 5). *Wing* (Figs. 5a and 7a–d): r3 pale with narrow dark hour-glass shape spot, incomplete, with posterior margin short, narrow, clearly not reaching M1 vein; cubital-anal fork pale (*n* = 58); dark spot in distal part of anal cell and an extra-dark spot present, smaller and close to the CuA2 vein, observed on only one wing for 17% of the specimens and on both wings for 52% of the specimens (*n* = 58); extra-dark spot rarely reaching CuA2 (2/58 wings) (Fig. 7c); sometimes, regular dark spot in anal cell absent (4/58 wings) (Fig. 7b); dark, rounded spot in distal part of m2 separated from the dark area on CuA1 (*n* = 58) (Fig. 7a-d) and the latter area rarely fused to the dark spot in m2 (3/58 wings); tips of the veins M1, M2, and CuA1 dark with sometimes centred by a pale spot in 71, 31, and 11%, respectively (*n* = 56) (see Table 4 for wing measurements). *Abdomen* (Fig. 5e): first abdominal tergite with 10.8 (6–14, *n* = 26) hairs. Spermathecae: 2 functional and 1 rudimentary; functional spermathecae ovoid, with short narrow pigmented neck, moderately sclerotized, equal in size (see measurements in Table 4); parallel sclerotized ring present. Sclerotization surrounding oviduct narrow, slightly parallel.

**Table 3:** Distribution of sensilla coeloconica along the flagellomeres. Data are provided for females and males of* C. bysta* n. sp. and *C. pulicaris *from Slovakia (SK) and France (FR); no males of *C. bysta* n. sp. were observed and measured in France

Species	Country	Flagellomere												
		1	2	3	4	5	6	7	8	9	10	11	12	13
Female														
*C. bysta* n. sp.	SK	6 (2–9)	0	0	0	0	0	0	0	1.4 (1–2)	1.7 (1–3)	1.9 (1–3)	2.1 (1–3)	2 (1–3)
C. bysta n. sp.	FR	5.6 (5–7)	0	0	0	0	0	0	0	1.5 (1–2)	1.5 (1–2)	1.8 (1–2)	2 (2–2)	1.9 (1–3)
*C. pulicaris*	SK	3.3 (3–4)	0	0	0	0	0	0	0	1.1 (0–2)	1.2 (1–2)	1	3.2 (2–5)	3.5 (2–5)
*C. pulicaris*	FR	4.7 (4–5)	0	0	0	0	0	0	0	1.1 (1–1)	1.3 (1–2)	1.4 (1–3)	2.3 (2–3)	2.8 (2–3)
Male														
*C. bysta* n. sp.	SK	2	0	0	0	0	0	0	0	0	0	1.2 (1–2)	2.6 (2–3)	2.9 (3–4)
*C. pulicaris*	SK	na	0	0	0	0	0	0	0	0	0	1 (0–2)	4.2 (1–7)	5.3 (2–11)
*C. pulicaris*	FR	na	0	0	0	0	0	0	0	0	0	1 (1–2)	4.5 (4–6)	5 (5–7)

**Table 4:** Measurements of the most important female body characters. The mean and the range of values (minimum-maximum) are given for each character with the exception of the data for *C. boyi* from Denmark (DK), taken from Nielsen et al. [23]. P/H ratio is given as the reverse of H/P ratio

Species	*C. bysta* n. sp.	*C. bysta* n. sp.	*C. pulicaris*	*C. pulicaris*	*C. boyi*
Country	SK	FR	SK	FR	DK
Wing length (mm)	1.5 (1.1–1.7)	1.4 (1.1–1.6)	1.6 (1.3–1.8)	1.6 (1.2–1.95)	1.6^a^
Wing ratio L/W	2.2 (1.9–2.3)	2.2 (2.1–2.3)	2.3 (2.2–2.4)	2.2 (2.0–2.2)	
Antennal ratio	1.03 (0.92–1.16)	1.09 (0.54–1.35)	1.03 (0.95–1.09)	1.05 (0.99–1.08)	1.3^a^
Ratio (R11/10)^*^	1.37 (1.26–1.50)	1.37 (1.25–1.45)	1.41 (1.14–1.54)	1.46 (1.30–1.54)	
AtR^***^	1.5 (1.15–1.87)	1.5 (1.2–1.8)	1.77 (1.56–2.08)	1.7 (1.5–1.8)	1.5 (1.3–1.7)
Palpal ratio^*^	2.7 (2.4–3.1)	2.7 (2.4–3.1)	2.4 (1.9–3.0)	2.3 (1.8–2.7)	2.9^a^
Proboscis lenght	209 (187.5–250)	206 (188–221)	228 (203–255)	229 (199–257)	218 (203–235)
P/H ratio^***^	0.8 (0.7–0.8)	0.8 (0.7–0.8)	0.8 (0.8–0.9)	0.9 (0.8–0.9)	
H/P ratio^***^	1.3 (1.2–1.4)	1.3 (1.3–1.4)	1.19 (1.1–1.2)	1.2 (1.1–1.2)	1.29^a^
Spermatheca I length (μm)	71 (51–81)	67 (49–76)	79 (71–90)	71 (65–79)	
Spermatheca I width (μm)	51.6 (46–58)	52 (48–59)	53 (47–64)	51 (47–61)	
Spermatheca lI length (μm)	67 (53–78)	67 (56–74)	64 (62–64)	69 (58–76)	
Spermatheca Il width (μm)	48.5 (44–55)	52 (47–58)	47 (39–58)	49 (45–54)	

Significant differences (***P < 0.001 and *P < 0.05) between *C. bysta* n. sp. and *C. pulicaris* are indicated considering the cumulative data from Slovakia (SK) and France (FR). No differences within the species and between the countries, or between *C. bysta* n. sp. and *C. boyi*, were observed. *Culicoides boyi* and *C. pulicaris* were significantly different for the AtR ratio (*P* < 0.05)

^a^Data from Nielsen et al. [23]

**Fig. 8:**
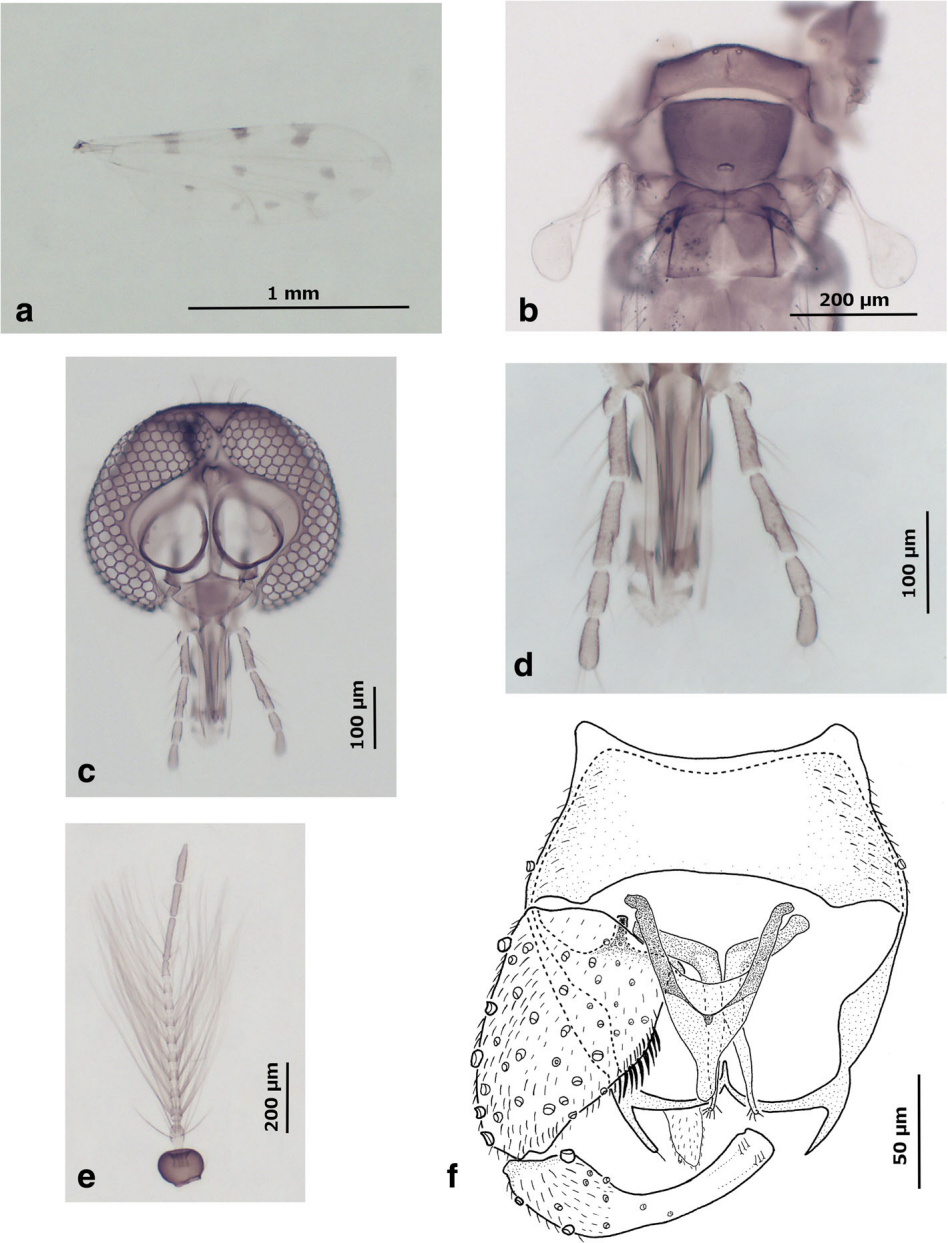
Illustrations of *C. bysta* n. sp. male. **a** Wing. **b** Scutellum, scutum and first abdominal segment. **c** Head. **d** Palpal segments. **e** Antenna. **f** Genitalia. *Scale-bars*: **a**, 1 mm; **b**, **e**, 200 μm; **c**, **d**, 100 μm; **f**, 50 μm

***Male*** *.* [Based on the holotype and 11 paratypes; Figs. 6d–f and 8.] *Head*: Eyes (Fig. 8c) bare, contiguous. Antenna (Fig. 8e, Table 3): sensilla coeloconica present on flagellomeres 1, 11–13 (Table 3); verticils of numerous sensilla chaetica from flagellomere 1 to 12, flagellomere 13 with 5.4 sensilla basally including 1 regularly present in median part of flagellomere, 2 sensilla on flagellomere 12, none on basal part of flagellomere 13 and 1 sensilla apically; blunt-tipped sensilla trichodea not swollen, distribution: 1.9 long on flagellomere 1, 2 long, and 1 short on flagellomeres 2 to 4, 1.1 long and 1.1 short on flagellomere 5, 1 long and 1 short on 6 and 7, 1 long and 0.4 short on 8 and absence of trichodea on flagellomeres 9 to 13 (*n* = 16); lengths of flagellomeres 1–13: 111, 47, 44, 44, 43, 43, 42, 40, 40, 50, 116, 93 and 115 (*n* = 15); total length of antenna 828 (*n* = 14); AR: 0.6 (*n* = 14); ratio between lengths of first long flagellomere and last short R11/10 = 2.3 (*n* = 14). Palpus (Fig. 8c, d) slender, palpal segment I with 1 long chaetica, segment II with 4 short chaeticae, segment III slightly swollen, carrying 4.6 short chaeticae, with multiple irregular pits located in third apical part of segment, segment IV with 3 short chaeticae, segment V without chaetica but with 5 apical bristles (*n* = 9); lengths of segments I-V: 67 (I + II), 67, 33 and 38 (*n* = 17); total length 205 (*n* = 17); PR: 4.2 (*n* = 18); R3/1 + 2: 1.01 (*n* = 16). Maxilla with several teeth-like structures; mandible without teeth. Cibarial and pharyngeal armature absent. *Thorax*: Legs (Fig. 6d–f) brownish, with usually pale bands, as in females; foreleg lacking spines on all tarsal segments (*n* = 22); lengths of fore femur, tibia, and tarsal segments: 429, 412, 229, 108, 67, 42 and 44 (*n* = 4); foreleg TR 2.1 (*n* = 4); middle leg with 2 spines distally on first tarsal segments, 2 on second, 1.9 on third, 1 on fourth (*n* = 22), lengths of middle femur, tibia, and tarsal segments: 550, 525, 281, 118, 71, 42 and 42 (*n* = 4); middle leg TR: 2.4 (*n* = 4); hind leg with hindtibial comb with 6.1 spines, lacking spines on all tarsal segments (*n* = 22); lengths of hind femur, tibia and tarsal segments: 488, 493, 255, 151, 84, 45 and 46 (*n* = 4); hind leg TR: 1.7 (*n* = 4). Wing pattern (Fig. 8a) similar to females; wing length × width 1360 × 460 (*n* = 9). *Abdomen* (Fig. 8b): First abdominal tergite with 12.3 lateral hairs (*n* = 15). Genitalia (Fig. 8f): Ninth sternite wide, with slight posteromedial indentation and sparse pubescence laterally; ventral membrane not spiculated; tergite 9 approximately as wide as long, with clear median cleft and long apicolateral processes (see measurements of apicolateral processes and related distances Dt and Db in Table 5); gonocoxite swollen in its basal part, middle part of internal edges lined with thick spines; ventral apodeme small, hook-shaped, dorsal apodeme cylindrical, robust; gonostylus barely longer than gonocoxite and twice as wide at basal edge as at apex, width of basal part reduced abruptly from 1/3 length to a parallel shape till the last 2/3 of gonostylus. Aedeagus (Fig. 8f) Y-shaped with round, short tip and long lateral arms, straight and curved at base; moderately sclerotized arch present in proximal part, where lateral arms join body of aedeagus. Parameres (Fig. 8f) separated, slender, becoming gradually very thin from proximal to distal part, tip of parameres terminating in fine pubescence.

**Table 5:** Measurements of male genitalia in micrometres presented as the mean followed by the range in parentheses

	N1	Lap	Wap	N2	Dt	Db
*C. bysta* n. sp.	24	22 (16–28)^***^	5.7 (3.6–7.2)	12	65 (54–72)^**^	77 (65–89)^***^
*C. pulicaris* ^a^	34	15 (8.5–22)^***^	4.9 (3.6–7.6	16	55 (45–67)^**^	59 (38–74)^***^
*C. pulicaris* SK	24	14 (8.5–22)	5.3 (3.6–7.6)	11	56 (45–67)	62 (38–74)
*C. pulicaris* FR	10	15 (9–20)	4.2 (3.6–5.4)	5	53 (47–61)	54 (49–59)

*Abbreviations*: *Lap* length of the apical processus, *Wap* width of the apical processus, *Dt* distance between the tips of the apical processes, *Db* distance between the bases of apical processes, *N1and N2* numbers of observations for Lap/Wap and Dt/Db, respectively

Significant differences (***P < 0.001 and **P < 0.01) between *C. bysta* n. sp. and *C. pulicaris* are indicated considering the cumulative data from Slovakia (SK) and France (FR); no significant differences were found between geographical populations of *C. pulicaris*

^a^The data from *C. pulicaris* were considered as a whole (*C. pulicaris*) or separately, regarding the origin in Slovakia (*C. pulicaris* SK) and France (*C. pulicaris* FR)

***Immatures*** **.** Unknown.

### Differential diagnosis

The combination of three characters of the wing pattern in females is discriminant to separate *C. bysta* n. sp. from *C. pulicaris* and *C. boyi*: (i) absence of a dark spot on the cubital-anal fork, (ii) a dark rounded spot in the distal part of m2, separated from the dark area on the CuA1 and, (iii) an incomplete and narrow hour-glass shape dark spot in r3 with the posterior margin short and narrow (Figs. 5a and 7a-d). In addition, several quantitative characters revealed significant differences, the most important being the Dios-ts and the ratio Dios-ts/Lios on the eyes (Table 6); the AtR ratio for *C. bysta* n. sp. is significantly smaller than that in *C. pulicaris* [1.50 (*n* = 52) *vs* 1.77 (*n* = 17); see Table 4]; the P/H ratio for *C. bysta* n. sp. is significantly smaller than that in *C. pulicaris* [0.77 (*n* = 26) *vs* 0.85 (*n* = 10); see Table 4]; the ratios of the first flagellomere are varied among *C. pulicaris*, *C. boyi* and *C. bysta* n. sp.: 1.52 ± 0.07 [22], 1.78 ± 0.07 [22], and 1.62 ± 0.1 (*n* = 33), respectively.

**Table 6:** Measurements of female eyes in micrometres. The mean and the range of values (minimum-maximum) are given for each character. Means indicated with the same letter (a-d) are not significantly different

Species	*n*	Lios	Dios-ts	Lts	Dios-ts/Lios
*C. bysta* n. sp.	18	30.5 (22.3–41.1)	25.9 (19.6–32)^a^	21.3 (14.8–27)	0.9 (0.5–1.2)^c^
*C. boyi*	5	25.5 (20.0–34.5)	27.6 (26.4–29.7)^a^	19.4 (17.1–22.6)	1.1 (0.8–1.5)^d^
*C. pulicaris*	4	29.9 (23–32.2)	21.3 (20.7–23)^b^	19.0 (16.1–23)	0.7 (0.6–0.9)^cd^

*Abbreviations*: *Lios* length of the inter-ocular suture of the joined eyes, *Dios-ts* distance between the inter-ocular suture and the transverse suture above the first inter-ocular seta, *Lts* length of the transverse suture, *Dios-ts/Lios* ratio of the above mentioned characters measured

Considering the lack of description of the male in *C. boyi*, the following diagnosis comments on the male of *C. bysta* are focused on comparison with *C. pulicaris*. The wing pattern of *C. bysta* n. sp. is similar to that in the female and may be used with confidence for the identification of males. In addition, the measurement of Db on the ninth tergite can discriminate *C. bysta* n. sp. from *C. pulicaris* with 77 (65–89, *n* = 12) μm and 59 (38–74, *n* = 8) μm, respectively, even though a small overlap exists there (Table 5). Differences are also observed on Lap and Dt but the overlap of measures is wider than with Db.

***Culicoides*** **(** ***Culicoides*** **)** ***boyi*** **Nielsen, Kristensen & Pape, 2015**

Syn. *Culicoides dk1* of Lassen et al. (2012) [18]

***Type-material examined*** **:** Denmark, Himmerland, Aalestrup, 56°40'5.13''N, 09°28'53.53''E, 9.ix.2008 (Paratype D, female), 22.vii.2009 (Paratypes E, F, 2 females); Himmerland, Nibe, 56°54'21.05''N, 09°37'23.90''E, 9.vii.2008 (Paratype G, female); Mors, Nykøbing Mors, 56°53'55.39''N, 08°48'41.65''E, 9.vii.2008 (Paratype H, female).

***Non-type material examined*** **:** Denmark, Zealand, Rønnede, 55°15'N, 12°01'E, 24–26.vi.2015 (2 females).

***Distribution*** **:** Denmark and recorded to France at the time of submission [22, 23, 34].

### Remarks

We evaluated the variations of the wing pattern of *C. boyi* and hereafter we present the summary of our observations (Fig. 7e, f): cubital-anal fork dark extended to at least a third of the vein (*n* = 14); a dark spot present in the distal part of the anal cell; a dark rounded spot present in the distal part of m2, usually connected from the dark area on the CuA1 (*n* = 14) with the dark spot and area rarely separated (4/14 wings); tips of the veins M1, M2, and CuA1 dark, with sometimes a slightly pale spot in M1 (10/14 wings). Based on these observations, we suggest to use first the wing pattern to discriminate between *C. bysta* n. sp. and *C. boyi* (see above).

## Discussion

The phylogenetic and morphological differences presented here justify the distinct status of *C. bysta* n. sp. within the Pulicaris group. The maximum interspecific genetic distance between the new species and *C. boyi* is low (7.6%), although greater than between the *C. selandicus* and the *C. kalix* (5.9%) [23]. For the most part, *cox*1 distances between species are usually found to be higher than 10% [15, 18, 19, 23, 35]. Thus, within the subgenus *Culicoides*, the lowest pairwise genetic distance was 12% between *C. fagineus* F1 and *C. subfagineus* (*s.s.*) in [15]. Moreover, the comparison of five closely related species within the subgenus *Avaritia* showed even lower genetic distance of 9.5% between *C. bolitinos* Meiswinkel, 1989 and *C. tutti-frutti* Meiswinkel, Cornet & Dyce, 2003 [34]*.* Although the genetic distance recorded between *C. bysta* n. sp. and *C. boyi* is low, the frequency distribution of pairwise genetic distances evidence a barcode gap between the intra- and the interspecific distances (Fig. 4). A similar graph was plotted to confirm the hypothesis of *C. scoticus* being a race of *C. obsoletus* [36]*.* As for the intraspecific distances, Pagès et al. [15] presented very low values (smaller than 0.6%) for all clades analyzed, including *C. newsteadi* N1. Moreover, the added specimen of *C. newsteadi* N1 sequenced in the previous study [19] exhibits a higher intraspecific genetic distance of 3.4% within the latter species. Thus the four existing and highly supported clades within *C. newsteadi* (*sensu lato*) indicate a clear need of an in-depth revision.

Genetically and morphologically, *C. boyi* is the closest species to *C. bysta* n. sp. and both exhibit morphological similarities to *C. pulicaris. Culicoides bysta* n. sp. can be distinguished from *C. boyi* and *C. pulicaris* by the combination of the following characters on the wing pattern: (i) the absence of a dark spot on the cubital-anal fork; (ii) the presence of a dark rounded spot in the distal part of m2 separated from the dark area on the CuA1 vein; and (iii) an incomplete and narrow hour-glass shape dark spot in r3 with short and narrow posterior margin. In addition to the ratio of the first flagellomere, the two newly evaluated characters are the most important for discrimination. Thus the females of *C. bysta* n. sp. possess a Dios-ts/Lios ratio significantly lower than that in *C. boyi* and higher than that in *C. pulicaris*. For males, Db measurements allow accurate discrimination between *C. bysta* n. sp. and *C. pulicaris.* Nevertheless, the male of *C. boyi* remains undescribed and the usefulness of Db as the discriminating character for males of *C. boyi* and *C. bysta* n. sp. should be investigated in future.

Detected in Slovakia, *C. bysta* n. sp. was recorded in areas from the eastern to the western parts of the country. This species appeared to be present in various environments such as farms with domestic ruminants or horses, in forests hosting game animals, as well as in zoological gardens and family houses with domestic animals and poultry. First recorded in Slovakia, *C. bysta* n. sp. was afterwards identified in France. This species appeared to not be as rare because the specimens were found in various localities in the north-east of France. During the preparation of the present description, additional specimens were also recorded in Bulgaria and Kosovo, indicating that *C. bysta* n. sp. may be widespread in Europe. As *C. pulicaris* is known to exhibit morphological variation [12–14], special attention should be paid in future studies on the Pulicaris group. For instance, at the time of submission of the present manuscript, an article was published with a new record of *C. boyi* for the fauna of France [34]. In the latter study, the genetic data fit perfectly with the data for *C. boyi* from Denmark, but morphologically the specimens from France exhibited variation. However, the combination of the above mentioned three characters may still be used for the accurate identification.

Several species morphologically similar to *C. pulicaris* and thus close to the newly described *C. bysta* n. sp. were considered to be competent vectors for BTV and SBV transmissions. As for BTV, *C. pulicaris* and *C. lupicaris* were involved in the transmission by virus isolation and RT-PCR, respectively [2, 7]*.* While both studies processed the pools of specimens identified by morphology, eventual presence of cryptic species within those pools remains possible. Similarly, a recent study implicated *C. punctatus* in the transmission of SBV by RT-PCR from pools of morphologically identified specimens [11]. To avoid the doubt which can be raised afterwards, studies dealing with *Culicoides* spp. should include molecular controls for the identification, such as (i) use of the diagnostic PCR tool for cryptic species within the subgenus *Culicoides*, for example [15], or (ii) sequencing of the barcode *cox*1 region as in a recent study [37] where *cox*1 was used to confirm the identification of specimens orally exposed to SBV. In the light of the increasing number of studies describing *Culicoides* spp. diversity, such as those providing evidence for the presence of cryptic species complexes and descriptions of new species, all studies using these midges as biological material should associate the molecular ID to their morphological ID. While none of the recent cryptic or new species have been involved in the arbovirus transmission so far, future studies focused on the evaluation of the role of *Culicoides* spp. in the transmission, taking into account the entire recently described diversity, may lead to overhaul the current knowledge of *Culicoides* transmitting diseases.

## Conclusions

We described here *C. bysta* n. sp. as a new species belonging to the Pulicaris group of the subgenus *Culicoides*. This species is closely related to the recently described *C. boyi* and to *C. pulicaris*. The phylogenetic analyses based on *cox*1 and the morphological differences justify *C. bysta* n. sp. as a distinct species. Female specimens of this new species described here can be distinguished by the wing pattern and by the ratio between two sutures on the joined eyes. This latter morphological character evaluated here for the first time, and the characters on the ninth tergite for males, are promising for species discrimination within the Pulicaris group. However, male of *C. boyi* remain unknown and comparison of males within the Pulicaris group requires further studies. The vector potential of the recently described species *C. boyi* and *C.bysta* n. sp. to transmit arboviruses, such as BTV and SBV, is unknown. The published data on vector implication of *C. pulicaris* in BTV transmission acquired prior the description of the two recently described species, *C. boyi* and *C. bysta* n. sp., should be re-evaluated in future.

## Abbreviations

BTV: Bluetongue virus
CDC: Centers for Disease Control
*cox*1: Cytochrome coxidase subunit 1
IPPTS: Institut de Parasitologie et de Pathologie Tropicale de Strasbourg
RT-PCR: Reverse transcriptase PCR
SBV: Schmallenberg virus
